# Combination of 10-hydroxy-decanoic acid and ZnO nanoparticles abrogates lead acetate-induced nephrotoxicity in rats: targeting oxidative stress and inflammatory signalling

**DOI:** 10.1186/s40360-025-00888-1

**Published:** 2025-03-25

**Authors:** Samar R. Saleh, Raheel G. Agwah, Samar S. Elblehi, Ahmed Z. Ghareeb, Doaa A. Ghareeb, Adham M. Maher

**Affiliations:** 1 Bio-Screening and Preclinical Trial Lab, Department of Biochemistry, Faculty of Science, Alexandria University, Alexandria, 21511 Egypt; 2 Department of Pathology, Faculty of Veterinary Medicine, Alexandria University, Alexandria, 22758 Egypt; 3 Center of Excellence for Drug Preclinical Studies (CE-DPS), Pharmaceutical and Fermentation Industry Development Center, City of Scientific Research & Technological Applications (SRTA-City), New Borg El Arab, Alexandria, Egypt; 4 Research Projects Unit, Pharos University in Alexandria, Canal El Mahmoudia Street, Beside Green Plaza Complex, Alexandria, 21648 Egypt

**Keywords:** Royal jelly, Antioxidants, Nrf2/HO-1 signalling, p-IKK/NF-κB/TNF-α signalling, Caspase-3/Bax/Bcl2 signalling, Apoptosis

## Abstract

Lead is a heavy metal contaminant that can cause significant alterations in renal structure and function, resulting in nephrotoxicity. The fatty acids of royal jelly exhibit immunoregulatory, anticancer, anti-inflammatory, and antioxidant properties, which have garnered significant interest. The most prevalent among them is 10-hydroxydecanoic acid (10-HDA). Zinc oxide nanoparticles (ZnONPs) demonstrate a renoprotective effect, likely due to their antioxidant, anti-inflammatory, and antiapoptotic properties. This study evaluated the therapeutic efficacy of 10-HDA and ZnONPs, administered either as monotherapy or in combination, against lead-induced nephrotoxicity. Male rats were orally administered lead acetate (PbAc) for three months, followed by the administration of 10-HDA and/or ZnONPs for one month. Exposure to PbAc resulted in elevated renal lead concentration, as well as increased serum levels of urea, creatinine, and cystatin C. The condition resulted in damage to the renal parenchyma, characterised by degenerative glomeruli and tubules, and exhibited the highest lesion score. Nrf2 and HO-1 exhibited reduced expression and diminished antioxidant enzyme levels subsequent to PbAc poisoning. Additionally, there was an increase in the inflammatory and apoptotic signalling through the p-IKK/NF-κB axis. The administration of 10-HDA and ZnONPs significantly decreased renal lead levels and improved antioxidant capacity. Moreover, renal inflammatory markers (TNF-α, p-IKK, IL-1β, IL-6, and IL-8) and proapoptotic indicators (Bax and Caspase-3) were significantly suppressed. The combined therapy demonstrated a synergistic effect (combination index < 1). In conclusion, the results indicated that 10-HDA and ZnONPs have the potential to be a supplement or even an effective treatment to alleviate the adverse effects of lead poisoning. This is potentially attributed to their potent ameliorative actions against oxidation, inflammation, and apoptosis.

**Supplementary Information:**

The online version contains supplementary material available at 10.1186/s40360-025-00888-1.

## Introduction

Heavy metal (HM) contamination represents a significant global public health concern [1]. This issue arises from the extensive disposal of these substances as industrial waste, which is non-biodegradable and persists in the environment for extended durations. The significant rise in their application across various industrial, agricultural, residential, and technological sectors has led to a notable increase in human poisoning incidents [2, 3].

Lead (Pb) is the most common heavy metal found in the environment. Metallic lead and its organic and inorganic salts are extensively used [4]. Lead possesses distinctive physical and chemical properties, such as its resistance to corrosion, softness, flexibility, and low conductivity. These properties render lead essential for the production of cookware, cosmetics, pharmaceuticals, paints, pipes, batteries, and book printing [5]. Lead exposure is associated with neurological, cardiovascular, renal, reproductive, and skeletal diseases [4]. Lead levels in the body can be assessed through blood sample analysis, microscopic examination of blood cell alterations, or X-ray imaging to identify dense line deletions in the bones [5]. Lead predominantly accumulates in the bones at elevated concentrations. However, the kidneys serve as the primary location for accumulation [6].

The kidney is essential for eliminating and accumulating lead, characterised by its complex structure and intricated physiological processes. The kidney is particularly susceptible to lead poisoning because of its distinct physiology and the toxicokinetics associated with lead exposure [7]. The proximal tubule cells eliminate lead, which causes renal tubular damage and renal failure by altering the cell’s physical absorptive activities and exhibiting mitochondrial dysfunction [8]. Oxidative damage arises from elevated levels of reactive oxygen species (ROS) induced by lead, which activates an inflammatory response in the kidney tissues [9, 10]. Therefore, lead exposure can increase the levels of the proinflammatory cytokines, including tumour necrosis factor (TNF-α), interleukin-1β (IL-1β), and interleukin-6 (IL-6) [11, 12].

Conventional treatments for lead intoxication include the administration of chelating agents, such as Meso 2,3-dimercaptosuccinic acid, D-penicillamine, sodium 2,3-dimercaptopropane-1-sulphonate, or calcium disodium ethylene diamine tetraacetic acid (CaNa_2_ EDTA). Most of these treatments exhibit limitations and side effects, including reduced levels of essential elements in the body and renal toxicity [13].

**Fig. 1:**
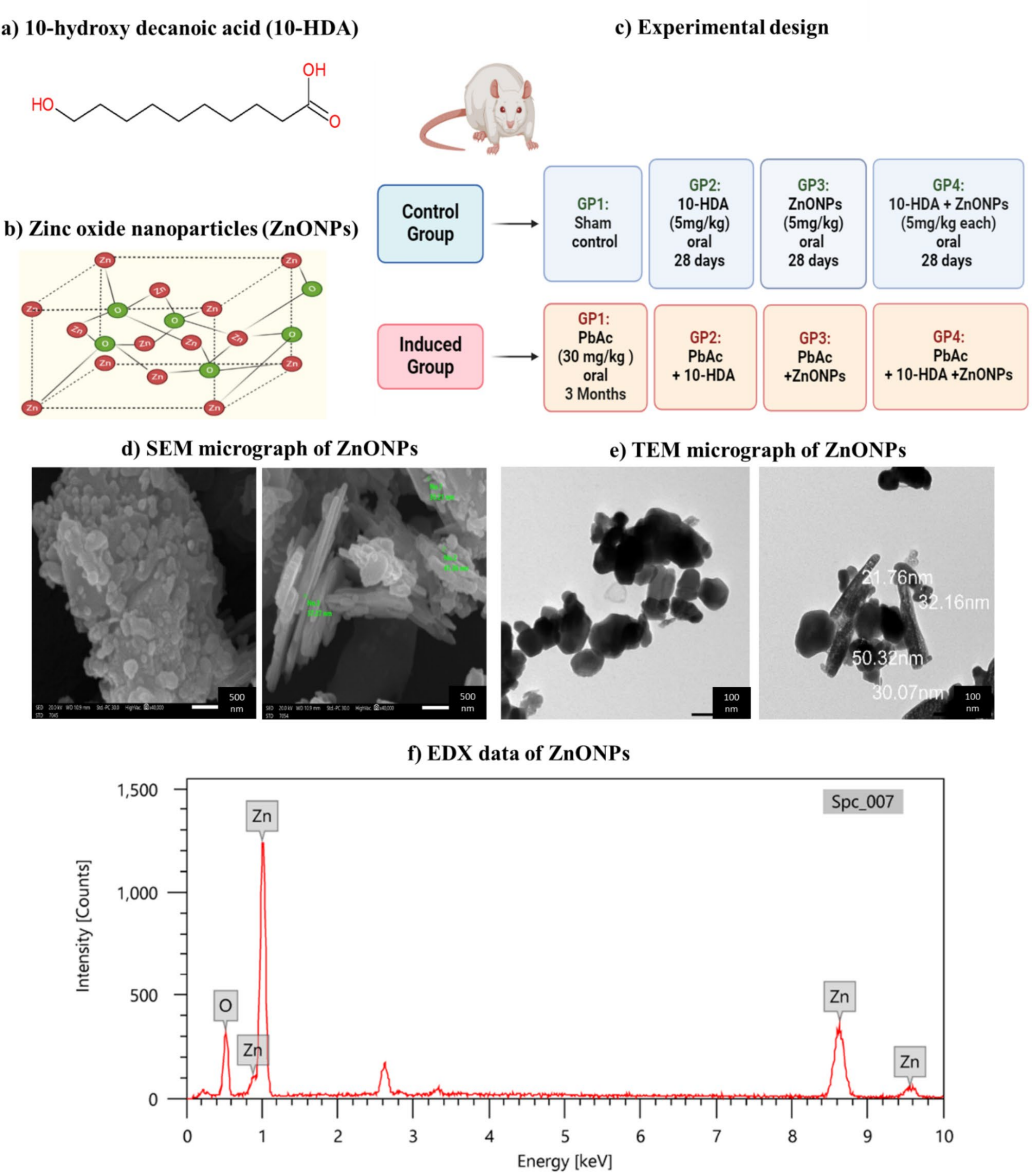
Chemical structure of 10-hydroxy decanoic acid (10-HDA) and Zinc oxide nanoparticles (ZnONPs), the experimental design, and the characterisation of ZnONPs. (**a**) 10-HDA is an unsaturated fatty acid with the chemical formula C_10_H_20_O_3_. Its structural representation features a hydroxyl group (-OH) attached to the 10th carbon of a decanoic acid chain. (**b**) ZnONPs are composed of zinc oxide with a nanoscale structure, exhibiting unique physical and chemical properties. Typically, these nanoparticles have a high surface-to-volume ratio and can be characterised using various techniques, including scanning electron microscopy (SEM), transmission electron microscopy (TEM), and energy-dispersive X-ray spectroscopy (EDX). (**c**) Regarding experimental design, the study aims to evaluate the therapeutic effects of 10-HDA and ZnONPs in a nephrotoxic rat’s model induced by lead acetate (PbAc). The experimental design shows the control groups (sham, 10-HDA, ZnONPs, and the mixed treatment) and the induced groups (PbAc, PbAc + 10-HDA, PbAc + ZnONPs, and the PbAc + mixed treatment). The treatments were continuous for 28 days at a dose of 5 mg/kg after an induction period of three months of lead acetate exposure. (**d**) SEM micrograph of ZnONPs -Magnification power x40.000- shows a spherical homogeneous shape of the nanoparticles, whereas the average particle size for the nanoparticle is about 40 nm. (**e**) TEM micrograph- Magnification power x50.000- revealed a hexagonal shape for ZnONPs. (**f**) The EDX data revealed the presence of two primary elements, Zn (82.43%) and O (17.57%), confirming the high purity of ZnONPs

The antioxidant properties of natural products are widely recognised, as they can function as scavengers of free radicals, thereby suppressing oxidative stress. These products may yield beneficial effects while minimising side effects [14]. Worker bees (Apis mellifera) produce royal jelly, a viscous substance secreted by their mandibular and hypopharyngeal glands. This substance has been widely used in various commercial products, nutritional supplements, and cosmetic formulations. Approximately 80–85% of the lipids in royal jelly are composed of fatty acids. This fatty acid, along with trans-10-hydroxy-2-decenoic acid (10-H2DA), constitutes 60–80% of the total fatty acids present in royal jelly [15, 16]. Moreover, 10-HDA exhibits unique therapeutic properties characterised by its immunomodulatory, antioxidant, anti-inflammatory, and oestrogenic [17–19]. The hydroxyl group at position 10 may correlate with its biological activities, linked to its distinctive structure (Fig. 1a).

Recent advancements in nanomedicine have enabled the utilisation of nanoparticles (NPs) for the diagnosis and treatment of various diseases. Therefore, a wide range of NPs is currently synthesised and undergoing comprehensive toxicity assessments [20]. Metal oxide nanoparticles are widely recognized as the most frequently utilised type of NPs and are frequently used in various applications [21, 22]. Zinc is an essential micronutrient necessary for the human body, serving as a cofactor for around 300 vital enzymes. Examples of these enzymes include extracellular and cytosolic superoxide dismutase (Zn/Cu SOD), which is crucial for the scavenging of ROS. Zinc plays a crucial role in inhibiting lipid peroxidation, thus protecting the body from damage induced by free radicals [23, 24]. The application of ZnONPs (Fig. 1b) remains controversial because of their ability to penetrate cellular membranes and interact with intracellular macromolecules, resulting in various therapeutic effects on particular organs. Additionally, ZnONPs have received approval from the Food and Drug Administration as a therapeutic option for cancer treatment [25, 26].

The current study aimed to evaluate the nephroprotective efficacy of 10-HDA and ZnONPs, both as monotherapy and in combination, against lead-induced nephrotoxicity. The efficacy of these treatments was assessed by examining the nuclear factor erythroid 2-related factor-2/heme oxygenase-1 (Nrf2/HO-1), p-IKK/NF-κB/TNF-α, and caspase-3/Bax/Bcl2 signalling pathways.

## Materials and methods

### Chemicals and reagents

Lead (II) acetate trihydrate (Pb (CH_3_CO_2_)_2_⋅3H_2_O) was obtained from Alpha Chemika Co. Andheri West, India. Zinc oxide nanoparticles (ZnO) were purchased from Nano Research Lab, Jamshedpur, India. The description on the package was as follows: size 30–50 nm, purity 99.9%. 10-HDA (99% purity) was provided from Sigma- Aldrich, St. Louis, USA. Kits for Urea and creatinine were acquired from Spectrum Diagnostics in Egypt. Tina-quant Cystatin C Gen. 2 assay kit was obtained from Roche Diagnostics, Belgium. GENEzol™ Reagent was acquired from Geneaid, Taiwan. TOPscript™ RT DryMIX (dT18/dN6) and TOPreal™ qPCR 2X PreMIX were obtained from Enzynomics, Korea. The primers were bought from Willowfort, UK. Rabbit monoclonal antibodies of Phospho-IKK α/β, Nrf2, HO-1, and β-actin (13E5) (#2697s, #82206s, #20733, and #7054, respectively) were purchased from Cell Signalling Technology, USA. Goat anti-Rabbit IgG, AP-linked Ab, was obtained from Cell Signalling Technology, USA. Analytical-grade chemicals and reagents were used throughout the study.

### Evaluation of zinc oxide nanoparticles

A dispersion of ZnONPs in ethanol was prepared and then ultrasonicated for 1 h. Following this, 4 µL of the ZnONPs was applied onto a Formvar membrane lining a copper grid.

A transmission electron microscope (TEM) (JEOL-JSM-1400 PLUS) was then utilised to inspect the ZnONPs applied to the grid. The nanostructure morphology and optical properties of ZnONPs were analyzed by coating the solid sample with a thin conductive layer of gold affixed to dual-sided carbon tape. Then, an SEM analysis was performed using JEOL JSM-IT 200 Scanning electron microscopy (SEM). Finally, the elemental constituents and purity of the ZnONPs were analyzed using the JOEL-IT 200 system of energy-dispersive X-ray spectroscopy (EDX) [27].

### Experimental animals and design

Eighty adult male Sprague Dawley rats (body weight: 150–200 g) were obtained from the animal facility at the Medical Research Institute, Alexandria University, Egypt. Rats were housed in polycarbonate cages (5 rats/cage).

Animal acclimatisation randomly assigned to a 12-hour light-dark cycle, a temperature of 22 ± 2.0 °C, and a humidity of 45–46%. The rats were allowed with unrestricted food and water. Animal handling was conducted in compliance with the ARRIVE guidelines. The experimental protocol was approved by the Institutional Animal Care and Use Committee at Alexandria University (AlexU-IACUC protocol no. AU 04 21 11 17 1 02).

Rats were aimlessly partitioned into two groups: the control and the induced group, as demonstrated in Fig. 1c. The control group was subdivided into **sham**, **10-HDA**,** ZnONPs**,** and comb (combined treatment: 10-HDA + ZnONPs)**. The induced group was subdivided into **PbAc**, **PbAc + 10-HDA**,** PbAc + ZnONPs**,** and PbAc + Comb**. Nephrotoxicity was induced by administering PbAc (30 mg/kg) daily via oral gavage for a duration of three months [28]. The weekly doses were determined according to the body weight of each rat. Distilled water was utilised to dissolve lead acetate. Subsequent to the induction period, the various treatment strategies outlined previously were implemented and continued for a duration of 28 days. Daily administration of 5 mg/kg of 10-HDA or ZnONPs was conducted at consistent times, with the appropriate amount dissolved in polyethylene glycol, considering the body weight of each rat [28].

#### Blood and tissue sampling and management

Rats were subjected to overnight fasting and then anaesthetised with 5% isoflurane for 3 min. The blood samples were collected and maintained for 15 min at room temperature, followed by centrifugation at 4 °C and 3000 rpm for 15 min to separate the serum. The serum was subsequently stored at -20 °C for later evaluation. Kidneys were promptly excised from all specimens. Kidney tissues were fixed using 10% neutral formalin for histopathological examination. A cold 0.9% NaCl solution was utilised to cleanse the kidneys for biochemical and molecular analyses, after which they were stored at -80 °C. The kidney tissues were homogenised using iced 0.1 M phosphate buffer saline (pH 7.4), then centrifuged for 15 min at 10,000 xg and 4 °C. The liquid phase was collected and stored at -80 °C for biochemical analysis.

### Estimation of renal lead content

The renal lead content was assessed through wet digestion methods. A sample weighing 200 to 400 mg was dried overnight in an oven at 120 °C and subsequently transferred to a cool muffle furnace, where it was heated at a rate of 50 °C per hour until it reached 450 °C. Samples are subsequently digested with concentrated nitric acid and then dehydrated in a muffle furnace at 450 °C for one hour.

The renal lead content was determined using flame atomic absorption spectrophotometry (Shimadzu model, AA-6650) at a wavelength of 283.3 nm, following the procedure outlined by Szkoda and Zmudzki [29].

### Serum and kidney biochemical analysis

**The serum urea level** was estimated using an enzymatic method involving the urease enzyme, which catalyzes the rapid hydrolysis of urea. The formed ammonia reacts with hypochlorite and phenol to give a blue dye read at 540 nm [30].

**The serum creatinine level** was determined based on its ability to react with picric acid, forming a coloured complex with an optical density read at 520 nm in an alkaline solution [31].

**The blood urea nitrogen (BUN) level** was assessed using the diacetyl monoxime technique [32]. Under acidic conditions, serum urea interacts with diacetyl monoxime in the presence of cadmium ion and thiosemicarbazide. The resulting rose-purple solution was quantified at 540 nm.

**The serum Cystatin C content** was assessed using a latex particle-enhanced immunoturbidimetry test following the method described by Newman and Thakkar [33]. The Tina-quant Cystatin C Gen. Two kit was utilised following the manufacturer’s instructions. Cystatin C interacts with latex particles that are coated with anti-cystatin C antibodies, leading to agglutination. A turbidimetric analysis was used to determine the aggregate at 516 nm. Serum urea, creatinine, and BUN were expressed in mg/dl, while Cystatin C level was expressed in mg/l.

**The renal superoxide dismutase (SOD)** activity was measured using the methodology established by Marklund and Marklund (1974). One unit of SOD activity corresponds to the quantity of enzyme required to produce a 50% decrease in the rate of pyrogallol auto-oxidation. SOD activity was expressed as U/mg protein. The total protein contents were measured according to Lowry and Rosebrough [34].

**The renal glutathione-S-transferase (GST)** activity was assessed using the protocol established by Habig and Pabst [35], which involves measuring the product glutathione nitrobenzyl at a wavelength of 310 nm. Glutathione nitrobenzyl is produced through glutathione S-transferase (GST) activity on its substrates, specifically P-nitrobenzyl chloride and reduced glutathione (GSH). The GST activity was expressed as U per mg of protein.

**The renal reduced glutathione (GSH) level** was quantified as mM/mg protein utilizing the procedure outlined by Ellman [36]. 2-nitro-5-thiobenzoic acid, characterised by its yellow colour, is generated through the interaction of GSH with DTNB. The colour developed can be assessed by measuring its absorbance at 412 nm to determine the concentration of glutathione (GSH).

**The measurement of malondialdehyde (MDA)** in kidney tissue was expressed as mM/mg protein. The procedure depends on the thiobarbituric acid reactivity upon heating to produce a pink-colored adduct. The optical density was measured at 532 nm, Tappel and Zalkin [37].

**The Nitric oxide (NO)** level in the kidney was reported as mM/mg protein. The evaluation utilised the methodology proposed by Montgomery and Dymock [38]. In acidic conditions, sulfanilamide reacts with nitrite to form a transient diazonium salt. A stable azo molecule is generated through the interaction of the intermediate with the coupling agent N-naphthyl-ethylenediamine. The product’s deep purple colour facilitates a sensitive nitrite assay and can be employed to quantify nitrite levels. The generated colour exhibits absorbance at 540 nm, which is utilised to determine NO concentration.

### Gene expression analysis: real‑time qRT‑PCR assay

In accordance with the manufacturer’s guidelines for GENEzol™ reagent, kidney tissue samples weighing 50–100 mg underwent total RNA extraction. A NanoDrop 2000 spectrophotometer (Thermo Scientific, USA) was calibrated to 260 nm and 280 nm to assess the quantity and purity of the isolated RNA by measuring the absorbance of the samples. Samples exhibiting A260/280 ratios of 1.8 or higher were utilised for subsequent analysis. For cDNA synthesis, 5 µg of RNA from each sample was subjected to treatment with TOPscript™ RT DryMIX (dT18/dN6). The qRT-PCR analysis was performed following this protocol: 1 µl of cDNA was mixed with 10 µl of TOPreal™ qPCR 2X PreMIX (SYBR Green with low ROX), 1 µl of forward primer, and 1 µl of reverse primer, bringing the total volume to 20 µl with RNase-free water. The CFX96TM Real-Time System (BIO-RAD, USA) was utilised to perform quantitative PCR by loading samples in duplicate and adhering to the following thermal protocol: denaturation for 12 min at 95 °C, followed by 45 cycles consisting of denaturation for 10 s at 95 °C, annealing for 30 s at 52 °C, and extension for 30 s at 72 °C. The quantification of target genes was performed by calculating the fold change via the comparative 2^-ΔΔCT^ method, utilising data from the sham control as the calibrator. GAPDH was used to normalise all values. Table 1 presents the primer sequences for the targeted genes and the corresponding annealing temperature.

**Table 1:** Data of gene primers examined in real-time PCR analysis

Name	Accession number	Forward primer	Reverse primer
IL-1β	NM_031512.2	GACTTCACCATGGAACCCGT	GGAGACTGCCCATTCTCGAC
IL-6	NM_012589.2	GCCAGAGTCATTCAGAGCAATA	GTTGGATGGTCTTGGTCCTTAG
IL-8	NM_017183.2	CATTAATATTTAACGATGTGGATGCG	GCCTACCATCTTTAAACTGCACAAT
TNF-α	NM_012675.3	ACACACGAGACGCTGAAGTA	GGAACAGTCTGGGAAGCTCT
Caspase-3	NM_012922.2	GTGGAACTGACGATGATATGGC	CGCAAAGTGACTGGATGAACC
Bax	NM_017059.2	AACTTCAACTGGGGCCGCGTGGTT	CATCTTCTTCCAGATGGTGAGCGAG
Bcl-2	NM_016993.2	GCAGCTTCTTTCCCCGGAAGGA	AGGTGCAGCTGACTGGACATCT
GAPDH	NM_017008.4	AGATCCACAACGGATACATT	TCCCTCAAGATTGTCAGCAA

IL-1β: Interleukin-1β; IL- 6: Interleukin-6; IL-8: Interleukin-8; TNF-α: Tumour necrosis factor alpha; Caspase-3: Cysteine–aspartic acid protease; Bax: Bcl-2 Associated X-protein; Bcl2: B-cell lymphoma 2; GAPDH: Glyceraldehyde phosphate dehydrogenase

### Western blotting analysis

The protein expression levels of phosphorylated inhibitor of nuclear factor-κB kinase (Phospho-IKK α/β), heme oxygenase-1 (HO-1), and nuclear factor erythroid 2-related factor-2 (Nrf2) were quantified through Western blotting analysis. The protocol developed by Mahmood and Yang [39] was applied using the protein extract derived from kidney tissue. SDS-PAGE was used to separate protein extract samples, and bands were then transferred to a nitrocellulose membrane. The membrane was then blocked with 5% bovine serum albumin and incubated with the primary antibody. Then, incubation with a secondary antibody was performed for immunoblotting. The membrane was exposed to an NBT/BCIP solution to view the protein bands. Quantity One software (Bio-Rad Laboratories, USA) measured the produced protein bands’ intensity and was calibrated using β-actin.

### Combination index (CI) analysis

The approach presented by Zhou and Li [40] was adopted to calculate the combination effect of 10-HDA and ZnONPs treatment. When compared to a single treatment, the combination may yield a better (synergistic), worse (antagonistic), or no different (additive) effect [41, 42]. The predictive value for the 10-HDA/ZnONPs combination was determined using the following equation:

$$\eqalign{{\rm{The}}\,{\rm{predicted}}\,{\rm{value}} & = {\mkern 1mu} \left( {{{10\, - \,{\rm{HDA}}\,{\rm{observed}}\,{\rm{value}}} \over {{\rm{Control}}\,{\rm{value}}}}} \right. \cr& \left. {\quad + \,{{{\rm{ZnONPs}}\,{\rm{observed}}\,{\rm{value}}} \over {{\rm{Control}}\,{\rm{value}}}}} \right) \cr& \quad \times \,{\rm{Control}}\,{\rm{value}} \cr} $$

The CI was calculated by exploiting the formula:

$$\:\text{C}\text{I}=\frac{\text{O}\text{b}\text{s}\text{e}\text{r}\text{v}\text{e}\text{d}\:\text{v}\text{a}\text{l}\text{u}\text{e}\:}{\text{P}\text{r}\text{e}\text{d}\text{i}\text{c}\text{t}\text{e}\text{d}\:\text{v}\text{a}\text{l}\text{u}\text{e}\:}$$

A CI value below, equal to, or larger than one indicates an antagonistic, additive, or synergistic effect, respectively.

### Histopathological analysis

Following necropsy, kidney tissue samples were rapidly fixed in 10% formalin (pH 7.4) with phosphate buffer for 24 h. The samples were then subjected to the conventional paraffin embedding procedure. Subsequently, 5-µm thickness slices were cut and placed on slides. The sections were then deparaffinated in xylene and rehydrated with ethanol at decreasing concentrations, followed by Haematoxylin and eosin (H&E) staining. A digital camera (EC3, Leica, Germany) was utilised to capture blind assessments of stained sections under a light microscope, with magnification settings of 100 and 400 (Leica, DM500).

The degree of histopathological lesions was assessed using a grading system for histological lesions. The various pathological lesions (vacuolated tubular epithelium, attenuated and necrotic renal tubular epithelium, dilated renal tubular lumen, interstitial inflammatory cell infiltrations, vascular congestion, glomerular necrosis, and lead deposition) were examined in each rat group. The analysis included five H&E-stained slides (one slide/rat) and ten random fields/slides, conducted in a blinded manner. The assessment of pathological lesions involved evaluating the percentage of tissue abnormality across the entire section. The percentage was used to categorize the lesions as follows: None: normal histology; Mild: 5–25% contribution of the examined area; Moderate: 26–50% involvement of the observed section; Severe: greater than 50% participation of the studied field. The total tissue damage scores were computed by adding the scores for each parameter [43].

### Statistical assessment

Statistical assessment was conducted using one-way analysis of variance (ANOVA) and post-hoc Tukey’s test. Data were presented as mean ± standard deviation (SD). Statical significance was set at p-value of < 0.05.

## Results

### Characterisation of ZnONPs

The characterisation of ZnONPs focuses their size, morphology, and surface properties, which are essential for comprehending their interactions in biological applications. The SEM image indicates that ZnONPs have an average particle size of about 40 nm and display a uniform spherical morphology. The TEM image indicated that the ZnONPs displayed a clear hexagonal morphology. EDX analysis revealed the presence of two main elements, Zinc (82.43%) and Oxygen (17.57%), thereby confirming the high purity of the ZnONPs (Fig. 1d-f).

### The effect of different treatments on kidney lead content

**Fig. 2:**
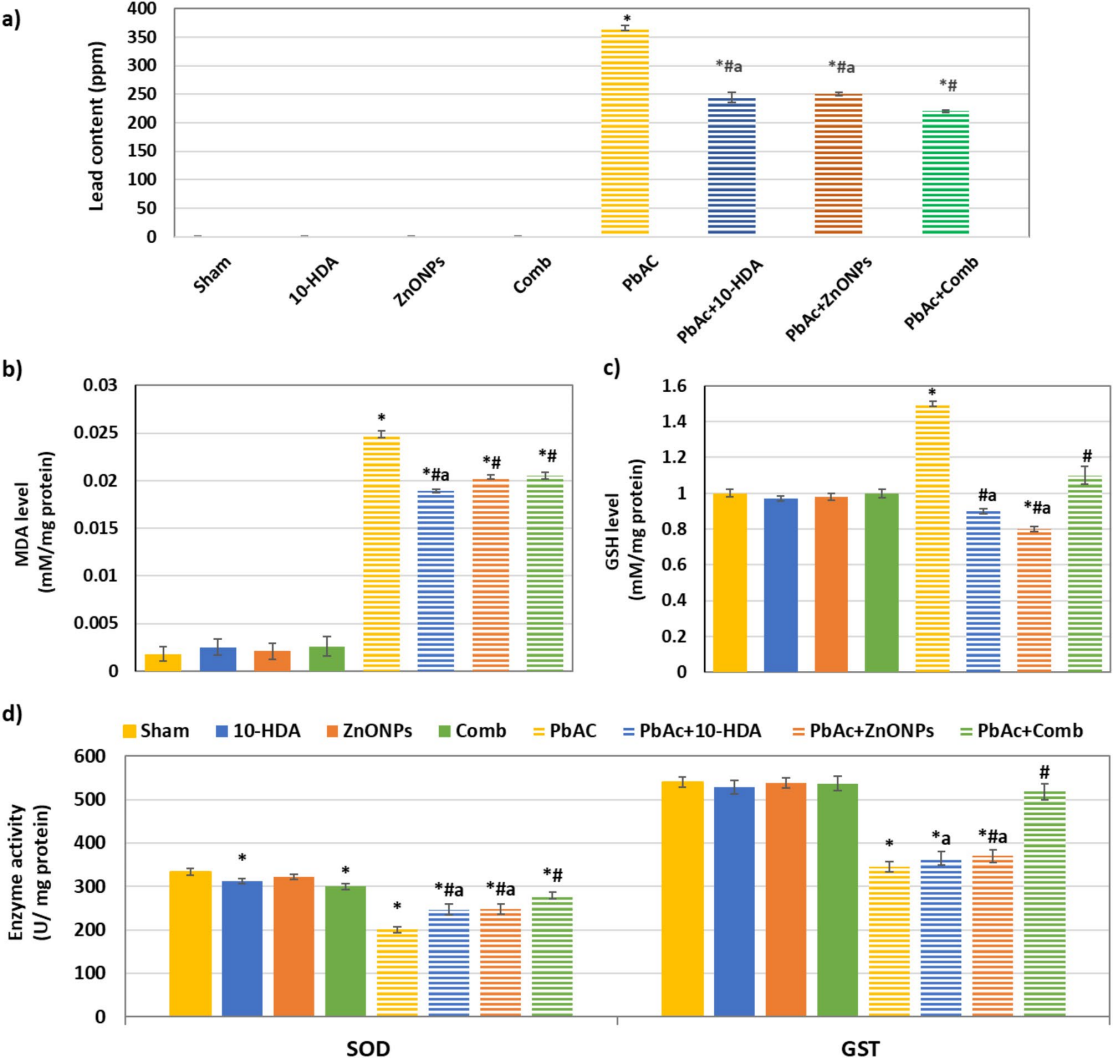
The effect of different treatments on kidney lead content, antioxidant, and lipid peroxidation markers. **a**) Kidney’s lead content, **b**) MDA level, **c**) GSH level, and **d**) SOD and GST activities. Administration of lead acetate significantly increased renal Pb content, MDA, and GSH levels. This was accompanied by a marked reduction in SOD and GST enzymatic activities compared to the sham group. Treatment with 10-HDA and ZnONPs, either individually or in combination, effectively mitigated these alterations. Notably, the combined treatment demonstrated superior efficacy in enhancing SOD and GST activities, as well as restoring GSH levels, compared to the individual treatments. Values are shown as mean ± SD (*n* = 10). *P < 0.05 vs. sham, ^#^*P* < 0.05 vs. PbAc-induced group, and ^a^*P* < 0.05 vs. PbAc + Comb

The lead content in the kidneys across the different experimental groups showed notable variations (Fig. 2a). A significant increase (*p* < 0.05) in lead kidney content was observed in the PbAc-induced rats compared to the sham rats. The administration of 10-HDA and ZnONPs, either individually or in combination, resulted in a significant reduction (*p* < 0.05) of kidney lead levels in rats subjected to PbAc induction. The combined treatment demonstrated the highest efficacy in lead detoxification. In all treated groups, kidney lead content remained significantly elevated (*p* < 0.05) compared to sham and control levels.

### The effect of different treatments on the serum kidney function parameters

Table 2 shows a significant increase (*p* < 0.05) in serum levels of blood urea nitrogen (BUN), urea, creatinine, and Cystatin C in the PbAc-induced group compared to the sham group. The individual treatments with 10-HDA and ZnONPs did not result in a significant reduction of serum parameters when compared to the PbAc-induced rats. The combination therapy (PbAc + Comb) resulted in a significant decrease (*p* < 0.05) in serum markers when compared to nephrotoxic rats induced by PbAc. The application of different treatment strategies to healthy animals did not lead to significant changes in kidney function parameters, in contrast to the sham group (Table 2).

**Table 2:** The impact of different treatments on the serum kidney function parameters

Investigational groups	BUN (mg/dl)	Urea (mg/dl)	Creatinine (mg/dl)	Cystatin C (mg/l)
Sham	11.6 ± 2.6	25.3 ± 2.5	0.40 ± 0.06	0.9 ± 0.27
10-HDA	11.5 ± 3.2	26.1 ± 2.9	0.38 ± 0.06	0.9 ± 0.26
ZnONPs	12.3 ± 1.9	24.7 ± 1.9	0.41 ± 0.08	0.8 ± 0.08
Comb	12.9 ± 1.7	26.1 ± 3.7	0.41 ± 0.01	0.9 ± 0.13
PbAc	**17.1 ± 2.7** *	**37.0 ± 1.8** *	1.10 ± 0.06*	2.1 ± 0.05*
PbAc + 10- HDA	16.8 ± 3.2*	36.0 ± 2.1* ^a^	0.97 ± 0.09*^a^	1.9 ± 0.5* ^a^
PbAc + ZnONPs	16.9 ± 1.1*	36.2 ± 1.9* ^a^	0.96 ± 0.09*^a^	2.2 ± 0.4*^a^
PbAc + Comb	14.6 ± 3.09*^#^	30.1 ± 2.1*^#^	0.72 ± 0.05*^#^	1.3 ± 0.2^#^

Values are shown as mean ± SD **(***n* = 10**).** *P < 0.05 vs. sham, ^#^*P* < 0.05 vs. PbAc-induced group, and ^a^*P* < 0.05 vs. PbAc + Comb

### The effect of different treatments on renal tissue antioxidant and lipid peroxidation markers

Rats administered PbAc exhibited notable changes in renal oxidative stress indices. This was supported by a significant increase (*p* < 0.05) in the levels of MDA and GSH in kidney tissues along with a notable reduction in the activity of SOD and GST (*p* < 0.05) in comparison to sham rats (Fig. 2b-d). The treatments with 10-HDA and ZnONPs, both individually and in combination, resulted in significant improvements in these variations (*p* < 0.05). The combined treatment demonstrated superior efficacy in increasing SOD and GST activities as well as GSH levels compared to the single treatment. No significant changes were observed in the levels of MDA and GSH, nor the activities of SOD and GST across the various control groups (Fig. 2b-d).

**Fig. 3:**
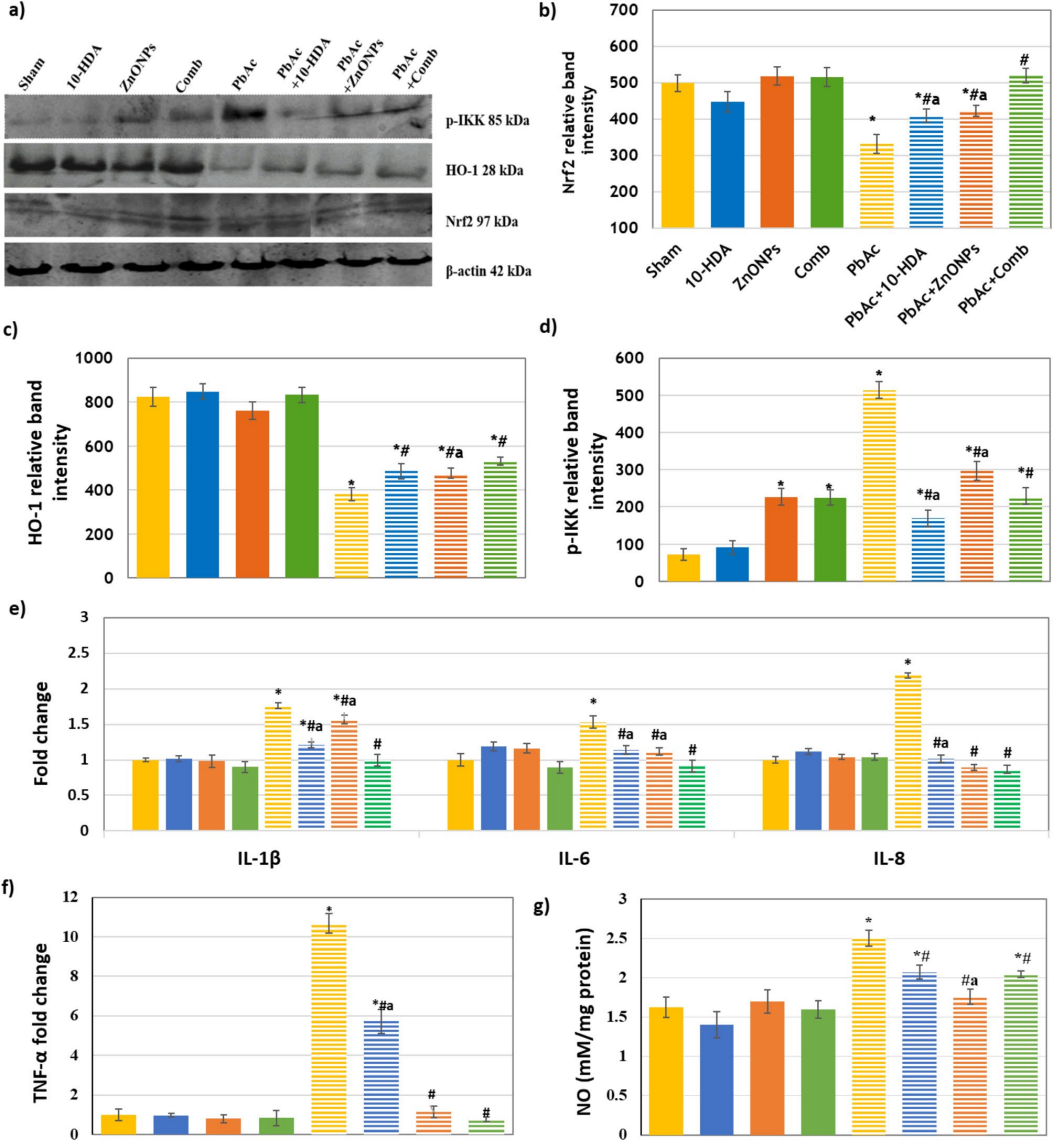
The effect of different treatments on the expression levels of renal inflammatory markers. **a**) Representative western blot images. **b**-**d**) Protein levels of renal Nrf2, HO-1, and p-IKK, respectively. **e** & **f**) Renal mRNA expression levels of IL-1β, IL-6, IL-8, and TNF-α. **g**) renal NO level. In the PbAc-induced group, a significant reduction in the protein levels of Nrf2 and HO-1 was observed compared to the sham group. Treatment with 10-HDA and ZnONPs, either individually or in combination, resulted in a marked increase in Nrf2 and HO-1 protein levels relative to the PbAc-induced rats. Furthermore, the combined treatment demonstrated greater efficacy in enhancing Nrf2 and HO-1 protein expression than the individual treatments. PbAc exposure triggered a strong inflammatory response in comparison to the sham group, as indicated by the elevated levels of kidney inflammatory mediators, including p-IKK protein level, the mRNA expression level of IL-1β, IL-6, IL-8, and TNF-α, as well as NO level. Treatment with 10-HDA and ZnONPs significantly reduced these proinflammatory markers in PbAc-induced rats. Values are shown as mean ± SD. *P < 0.05 vs. sham, ^#^*P* < 0.05 vs. PbAc-induced group, and ^a^*P* < 0.05 vs. PbAc + Comb

The expression levels of Nrf2 and HO-1 proteins in renal tissues across the various experimental groups are presented in Fig. 3a-c. A notable reduction (*P* < 0.05) in the protein levels of Nrf2 and HO-1 was observed in the PbAc-induced group relative to the sham group. In contrast, the levels of Nrf2 and HO-1 proteins showed a significant increase (*P* < 0.05) following the mono and combined treatments of 10-HDA and ZnONPs in PbAc-induced rats (Fig. 3a-c). The combined treatment demonstrated greater efficacy in enhancing the protein levels of Nrf2 and HO-1 relative to the individual treatments. Post-treatment assessments revealed that Nrf2 and HO-1 protein levels remained significantly lower (*P* < 0.05) than those observed in the sham rats. The control groups treated with 10-HDA and/or ZnONPs exhibited no significant differences in Nrf2 and HO-1 levels compared to the sham control group (Fig. 3a-c).

### The effect of different treatments on renal tissue inflammatory markers

Figure 3d-g demonstrates that the group exposed to PbAc nephrotoxicity exhibited a significant inflammatory response (*p* < 0.05) compared to the sham group. The findings indicate that the levels of kidney inflammatory mediators, including NO, p-IKK protein, and mRNA expression of TNF-α, IL-1β, IL-6, and IL-8, were significantly altered. Treatment with 10-HDA and ZnONPs significantly reduced proinflammatory markers (*p* < 0.05) in rats exposed to PbAc. The combined treatment demonstrated greater efficacy in reducing the majority of these parameter levels compared to the single treatment. The parameters of 10-HDA, ZnONPs, and the combined control groups exhibited no significant differences compared to the sham control group. The protein levels of P-IKK in normal rats administered ZnONPs, both mono and in combination, were significantly higher compared to the sham control group (*p* < 0.05), as demonstrated in (Fig. 3).

### The effect of different treatments on renal tissue apoptotic and survival responses

**Fig. 4:**
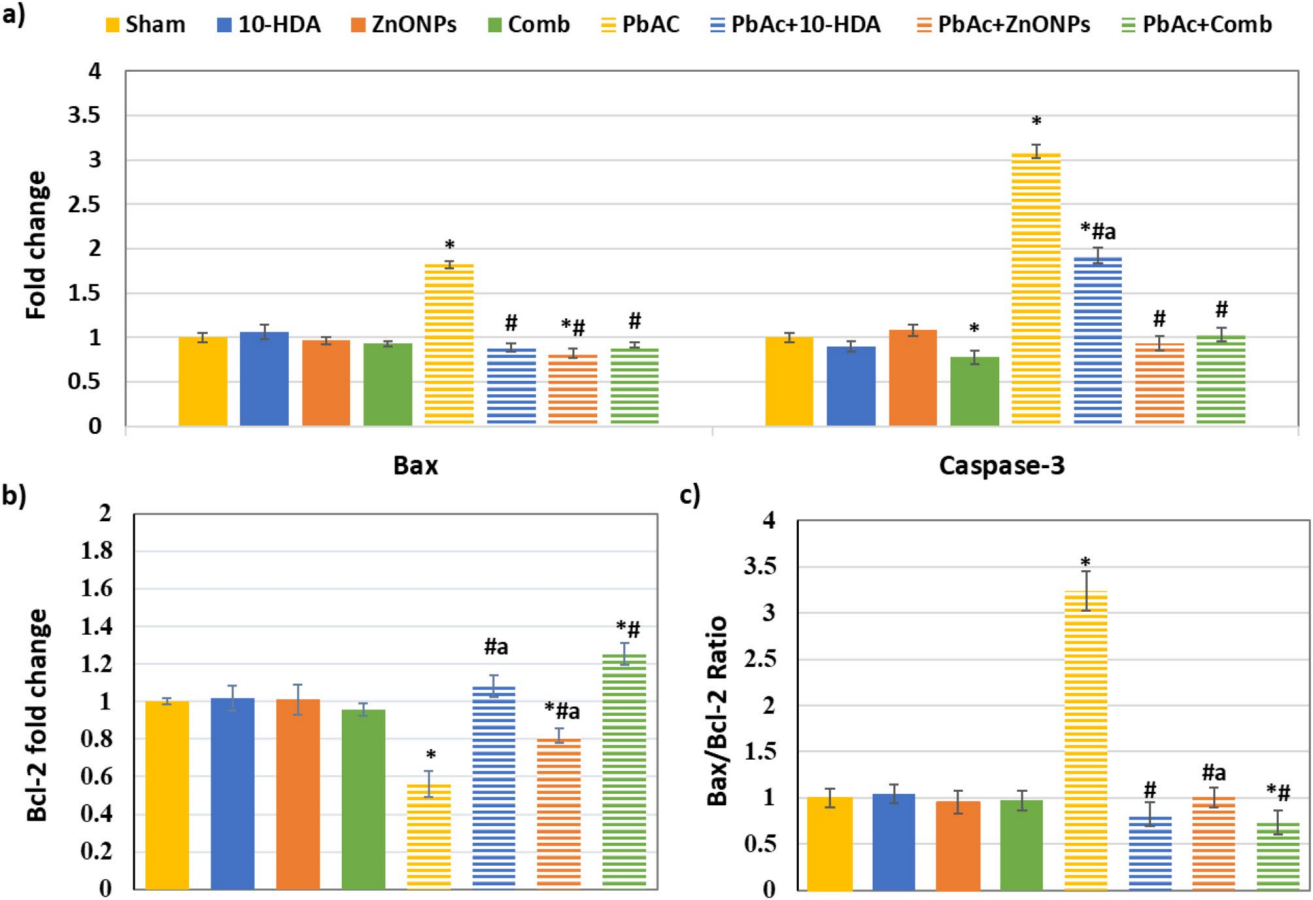
The effect of different treatments on the expression levels of renal tissue apoptotic and survival responses. **a** & **b**) Renal mRNA expression levels of Bax, caspase-3, and Bcl-2, respectively. **c**) Bax/Bcl-2 ratio. Exposure to PbAc resulted in a significant upregulation in the mRNA expression levels of Bax and Caspase-3 and an increased Bax/Bcl-2 ratio. Additionally, a substantial decrease in Bcl-2 expression was observed compared to the sham group. The treatment with 10-HDA, ZnONPs, and their combination led to a marked restoration of Bax and Caspase-3 expression. These interventions also significantly improved Bcl-2 expression and the Bax/Bcl-2 ratio. Notably, the combined treatment exhibited a more pronounced enhancement of Bcl-2 expression compared to the individual treatments. The results are expressed as mean ± SD. *P < 0.05 vs. sham, ^#^*P* < 0.05 vs. PbAc-induced group, and ^a^*P* < 0.05 vs. PbAc + Comb

The RT-PCR results displayed in Fig. 4 demonstrate that long-term administration of PbAc for three months induced significant (*P* < 0.05) elevations in the mRNA expression levels of Bax and Caspase-3 and Bax/Bcl-2 ratio. There was also a notable drop in the Bcl-2 expression level compared to the sham group. Regarding PbAc-induced rats, 10-HDA, ZnONPs, and combined treatments resulted in a significant recovery (*P* < 0.05) in the expression levels of Bax and Caspase-3. Furthermore, these treatments significantly enhanced the Bcl-2 expression level and attenuated Bax/Bcl-2 ratio (Fig. 4). The combined treatment also significantly enhanced the Bcl-2 expression level compared to the single treatment. Regarding 10-HDA and/or ZnONPs control groups, no significant variations were observed in most of these apoptotic and survival markers compared to the sham control values (Fig. 4).

### Synergistic combination of 10-HDA with ZnONPs against lead acetate-induced nephrotoxicity

Currently, drug combinations are increasingly utilised in the treatment of various serious diseases, such as cancer and infectious diseases, owing to their therapeutic advantages. This method can improve treatment effectiveness and decrease the risk of resistance, establishing it as a crucial strategy in contemporary medicine [44, 45]. Combination index methods are commonly employed to assess the efficacy of drug combinations. The Chou-Talalay method is a widely acknowledged approach that utilises the median-effect equation for quantitative analysis of drug interactions. This method categorizes interactions as synergistic (CI < 1), additive (CI = 1), or antagonistic (CI > 1) using the combination index equation [46]. The combination index (CI) values of 10-HDA and ZnONPs being less than 1 indicate a synergistic effect in the treatment of lead-induced kidney toxicity (Table 3). This suggests that the combined treatment is more effective than either alone in improving kidney function and reducing oxidative stress, inflammation, and apoptosis markers.

**Table 3:** The combination index values of 10-HDA and ZnONPs, including kidney lead content, kidney function tests, and the markers of oxidative stress, inflammation, and apoptosis

	CI value	Effect
Tissue lead content (ppm)	0.44 ± 0.008	Synergism
Kidney function parameters		
BUN (mg/dl)	0.43 ± 0.019	Synergism
Urea (mg/dl)	0.41 ± 0.020	Synergism
Creatinine (mg/dl)	0.37 ± 0.005	Synergism
Cystatin C (mg/l)	0.30 ± 0.04	Synergism
Oxidative stress indices		
MDA (mM/mg protein)	0.52 ± 0.086	Synergism
GSH (mM/mg protein)	0.63 ± 0.022	Synergism
GST (U/mg protein)	0.72 ± 0.015	Synergism
SOD (U/mg protein)	0.56 ± 0.022	Synergism
Nrf2 (relative band intensity)	0.564 ± 0.025	Synergism
HO-1 (relative band intensity)	0.55 ± 0.006	Synergism
Inflammatory mediators		
TNF-α (Fold change)	0.10 ± 0.002	Synergism
IL-1β (Fold change)	0.33 ± 0.023	Synergism
IL-6 (Fold change)	0.37 ± 0.022	Synergism
IL-8 (Fold change)	0.45 ± 0.046	Synergism
p-IKK (relative band intesity)	0.492 ± 0.031	Synergism
NO (mM/mg protein)	0.53 ± 0.019	Synergism
Apoptotic markers		
Bcl-2 (Fold change)	0.60 ± 0.014	Synergistic
Caspase − 3 (Fold change)	0.33 ± 0.03	Synergistic
Bax (Fold change)	0.47 ± 0.03	Synergistic

A CI value of < 1 indicates a synergistic effect, > 1 indicates an antagonistic effect, and = 1 indicates an additive effect. The values are expressed as means ± SD.

### Histological examination

**Fig. 5:**
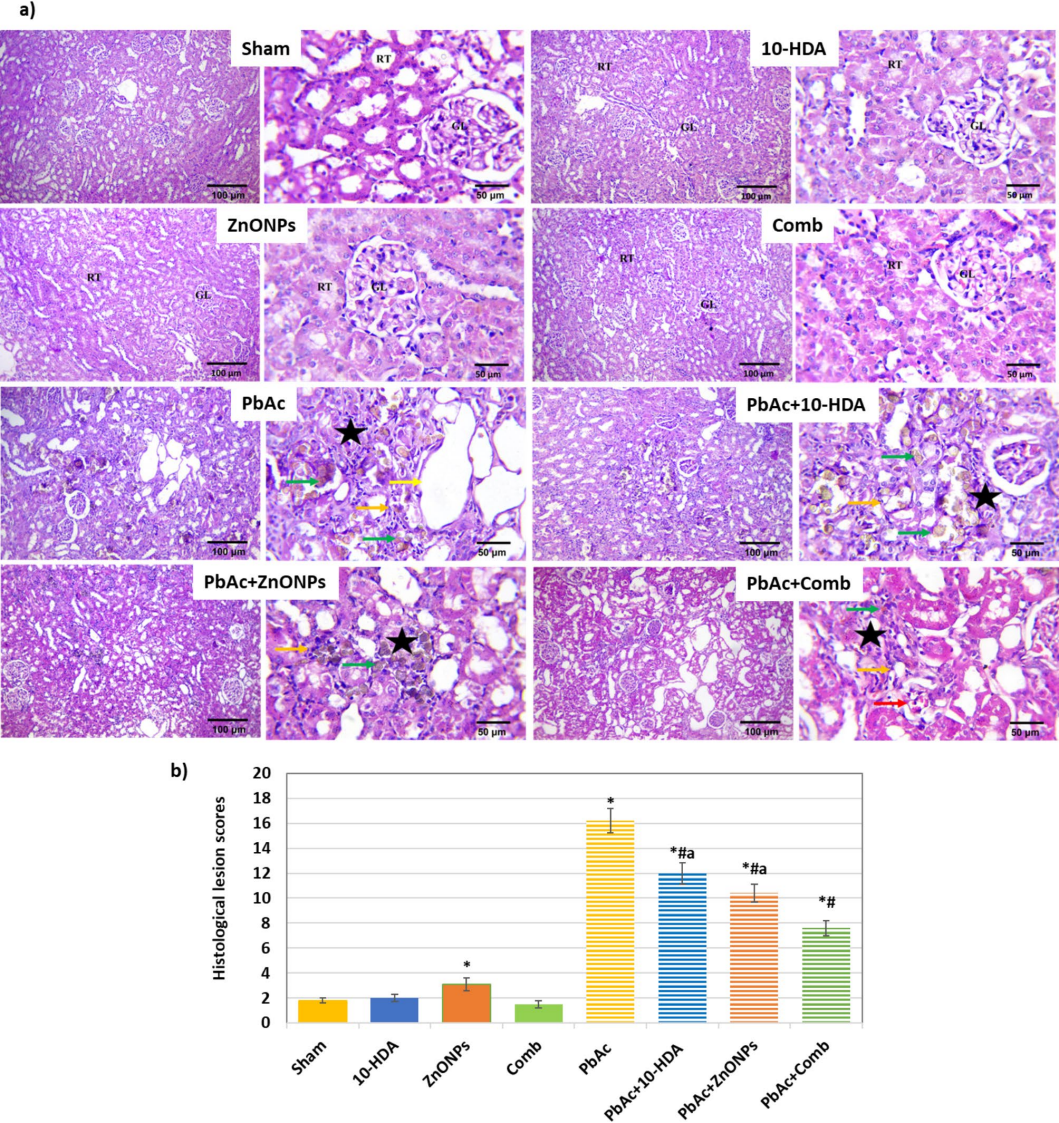
Histopathologic changes and quantification of the histological lesion score in renal tissue sections with H&E-staining across various groups. **a**) Representative photomicrographs demonstrating the histopathological alterations (×100 & ×400). Normal histoarchitecture of glomeruli (GL), renal tubule (RT), attenuated and necrotic tubular epithelium (orange arrow), dilatation of the renal tubular lumen (yellow arrow), interstitial mononuclear cell infiltrations (star) congested blood vessels (red arrow), deposition of lead particles (green arrow). **b**) Quantification of the histological lesion score in the studied groups of rats. The pbAc-induced group exhibited a higher lesion score than the sham group, while the combined treatment demonstrated promising efficacy in reducing the lesion score. The values are presented as mean ± SD. *P < 0.05 vs. sham, ^#^*P* < 0.05 vs. PbAc-induced group, and ^a^*P* < 0.05 vs. PbAc + Comb

Figure 5a shows that the kidney tissue images from the sham group and those treated with 10-HDA and ZnONPs, either alone or in combination, exhibited genuine morphological renal parenchyma characterised by well-defined organisation of glomeruli and tubules. The renal tubules in the PbAc nephrotoxicity-induced group exhibited significant degenerative changes (Fig. 5a). The observed changes are characterised by moderate swelling of the tubular epithelial cells, leading to enlargement towards the tubular lumen and a narrow star-shaped lumen. Granular and foamy cytoplasm is also observed in the tubular epithelium cells. Other tubular epithelial cells displayed a moderate level of cytoplasmic vacuolation. Additionally, necrosis and tubular epithelium attenuation were observed. The tubular lumen displayed widened Bowman’s space with necrotic capillary tufts. Also, dark eosinophilic necrotic debris can be noticed in the tubular lumen. Regarding the renal parenchyma, tubulointerstitial nephritis is noticeable. Mononuclear cell infiltrations and lead particles deposition can also be seen in the renal parenchyma. Furthermore, glomerular and vascular congestion associated with focal areas of hemorrhage were also seen. Similarly, this group demonstrated the highest lesion scores (Fig. 5b). Conversely, 10-HDA, ZnONPs, and combined treatments ameliorated the previous alterations and resulted in renal tissue histoarchitecture improvements. The combined treatment group demonstrated the most significant improvement. Nevertheless, they exhibited similarity to the control limits, as depicted in (Fig. 5a & b).

## Discussion

The extensive use of heavy metals has resulted in the contamination of environmental matrices, presenting significant health risks [47]. HMs such as iron, manganese, zinc, and copper are vital for physiological processes. However, the physiological function of metals such as arsenic, cadmium, and lead remains unknown. Despite their low concentrations, the renal capacity to reabsorb and accumulate certain divalent metals renders them significantly harmful to the kidney [6, 48]. The severity of renal damage is contingent upon the type, dosage, duration of exposure, and route of administration [49, 50].

Lead, a prevalent pollutant in the environment, can substitute for biological metals because of its greater affinity for various ligands, including zinc-finger transcription factors, proteins containing sulfur-rich amino acids, and the metal-binding protein metallothionein [51]. The kidney is a vibrant, metabolically active tissue that engages in numerous metabolic processes. Lead accumulation in the kidney may lead to both organ and systemic damage [52]. The renal ability to reabsorb and accumulate specific divalent metals, despite their low concentrations, presents considerable risk to the kidney [6, 53, 54]. The findings indicated that exposure to PbAc led to an accumulation of lead in renal tissue, signifying the presence of lead poisoning and its deposition in this organ. Additionally, Pb accumulation correlated with increased serum levels of urea, creatinine, BUN, and Cystatin C, suggesting impaired renal function and nephrotoxicity [55]. Cystatin C functions as a proteinase inhibitor and is continuously produced by all nucleated cells. The low molecular weight (Mr = 13 kDa) enables free filtration by the renal glomeruli. The level of serum Cystatin C is regulated by the glomerular filtration rate (GFR), making it a suitable endogenous marker of GFR and an indicator of nephrotoxic and ischemic kidney injury [56]. Therefore, the elevated levels observed following PbAc intoxication were due to renal parenchymal damage and decreased GFR. To address the limitations and adverse effects associated with conventional treatments commonly used to treat lead toxicity, scientists are constantly searching for solutions that are often associated with limitations and adverse effects [13]. The administration of antioxidants as a supplement may reduce lead accumulation in renal tissue by binding to the metal and promoting its elimination [6, 57]. This study investigated the renoprotective effects of combining 10-HDA and ZnONPs in mitigating PbAc-induced renal injury in rats. Additionally, 10-HDA demonstrates antioxidant and anti-inflammatory properties [58, 59] and its distinct characteristics enable interaction with biological macromolecules, resulting in various therapeutic effects [21, 22]. Various underlying mechanisms were examined, including oxidative stress, Nrf2-/HO-1 pathways, as well as apoptotic and inflammatory pathways.

In the current study, the administration of 10-HDA and ZnONPs led to a decrease in the renal Pb content, indicating improved kidney clearance. Both 10-HDA and ZnONPs potentially exhibit chelating activity. The combined treatment demonstrated enhanced efficacy in eliminating HMs. Nonetheless, only the combined treatment caused a significant improvement in the kidney function parameters, revealing the nephroprotective properties that can be attributed to the synergistic activity of these components. Our results agree with those of Barakat, Barakat [26], Almeer, AlBasher [59], and Alaraj [60], who demonstrated the nephroprotective effect and the attenuation of the induced nephrotoxicity by 10-HDA (royal jelly) or ZnONPs.

Oxidative stress serves as a primary factor in the progression of nephrotoxicity due to its sustained presence in renal tissue. Lead (Pb) induces oxidative stress by increasing the levels of ROS and simultaneously reducing the antioxidant defence mechanisms [61, 62]. This study demonstrates that PbAc intoxication disrupts the renal oxidative and antioxidative balance, evidenced by elevated levels of oxidants such as NO and MDA, a primary byproduct of lipid peroxidation. Furthermore, PbAc exposure reduced the activities of the critical antioxidant enzymes, SOD and GST and led to a downregulation in the protein expression levels of renal Nrf2 and HO-1. The impaired activity of these enzymes is likely caused by direct inhibition from Pb^2+^ through cation interaction or reduced enzyme synthesis [63]. For instance, Pb^2+^ can diminish the activity of enzymatic antioxidants by competing with metals that function as cofactors or interacting with sulfhydryl groups in enzymatic and nonenzymatic antioxidant molecules [61]. The Nrf2/HO-1 pathway protects cells and cellular components from oxidative stress-induced damage. Nrf2 exhibits multifunctional nuclear potency by activating the expression of genes involved in detoxification, the antioxidant system, and the resolution of inflammation. Nrf2 regulates the transcription of multiple antioxidant enzymes, including HO-1, SOD, GST, glutathione peroxidase, and γ-glutamyl cysteine synthetase [64–66]. Therefore, the current study’s deactivation of the antioxidant enzymes can be accredited to the suppression of Nrf2 and HO-1 genes following PbAc toxicity. These findings align with previous reports [6, 9, 67–71]. Our findings also demonstrate that exposure to PbAc elevated the levels of GSH, a molecule known for its antioxidant properties. This elevation aligns with the findings of a previous study on HM cadmium, utilised to induce testicular oxidative stress. The authors proposed that cadmium may conjugate with GSH, thereby preventing its negative inhibition of γ-glutamyl cysteine synthetase, a key enzyme in GSH biosynthesis. This leads to an elevation of GSH levels in the tissue [72]. Lead may induce a comparable effect on γ-glutamyl cysteine synthetase, resulting in elevated GSH levels in renal tissue.

The administration of 10-HDA and ZnONPs after PbAc exposure restored redox homeostasis in renal tissue. The findings were evidenced by reduced levels of MDA, GSH, and NO, elevated activities of antioxidant enzymes (SOD and GST), and enhanced expression of Nrf2 and HO-1. Nrf2 is recognised for its role in regulating the expression of enzymes associated with phase II detoxification, metabolism, and antioxidation. Previous research has shown that the expression levels of GST and HO-1 are markedly reduced in Nrf2-deficient mice [73, 74]. The supplementation of 10-HDA and ZnONPs resulted in the most significant upregulation of Nrf2 and HO-1 levels, as well as increased GST activity. The findings align with the minimal Pb concentration observed in the kidney. The administration of ZnONPs was previously found to reduce MDA levels and enhance the expression of antioxidant enzymes (SOD and catalase) and genes (Nrf2 and HO-1) in a rat model of adenine-induced renal oxidative damage [75]. Moreover, previous studies revealed that pretreatment with 10-HDA (royal jelly) enhanced Nrf2/HO-1 signalling in cadmium-induced nephrotoxicity in male mice [59]. The current experiment demonstrates that the upregulation of Nrf2 and HO-1 expression after supplementation with 10-HDA and ZnONPs suggests their role in reducing PbAc-induced oxidative accumulation in renal tissue through the activation of the Nrf2/HO-1 defence pathway. This observation may elucidate the detected improved levels of antioxidant defensive mediators in response to the combined treatment. Additionally, these molecules can be interpreted in terms of their antioxidant properties. Monitoring cytokine levels and other inflammatory mediators has become increasingly important, as alterations in their expression profiles are linked to the pathophysiology of lead exposure and clinical findings [6]. Lead activates the NF-κB pathway, which triggers inflammatory and immune responses by generating proinflammatory cytokines. Furthermore, this inflammatory response is complemented by the production of ROS, leading to subsequent damage to tissues and cells [11, 12]. In the cytoplasm of non-stimulated cells, the heterodimeric transcription factor NF-κB is conserved by the constrain of inhibitory proteins (IkBs). Upon stimulation, proinflammatory stimuli as TNF-α and IL-1β trigger the multi-subunit protein kinase IkB kinase (IKK) activation and phosphorylation. Consequently, IKK phosphorylates the regulatory domain of IkBs, resulting in its polyubiquitination and subsequent rapid degradation. NF-κB dimers enter the nucleus and start the transcription of TNF-α, IL-1β, and IL-6 [12, 76]. Moreover, the surged activity of inducible NO synthase (iNOS) and the fired generation of toxic NO is also mediated through the activation of the NF-κB signalling pathway [77, 78]. Data indicated that exposure to PbAc elevated p-IKK and the levels of proinflammatory cytokines (TNF-α, IL-1β, IL-6, IL-8, and NO) in kidney tissue. The findings align with those reported by Salama, Arab [79], Offor, Mbagwu [80], Albarakati, Baty [9], Bhattacharjee, Kulkarni [10], and Kucukler, Benzer [81]. Therefore, inhibiting the synthesis of proinflammatory cytokines may serve as a viable approach to alleviate PbAc-induced nephrotoxicity. Both 10-HDA and ZnONPs treatments effectively inhibited the significant elevation of p-IKK and proinflammatory cytokines in renal tissue. The findings revealed the anti-inflammatory potential of these molecules by inhibiting p-IKK/NF-κB activation and obstructing the release of inflammatory mediators [82]. Multiple previous studies have demonstrated the anti-inflammatory effects of 10-HDA (royal jelly) through the inhibition of NF-κB signalling [58, 59, 83, 84]. Earlier studies demonstrated that ZnONPs expressed strong anti-inflammatory effects by hindering tissue NF-κB levels while lowering mRNA and protein levels of iNOS, TNF-α, IL-1β, IL-6 [85–88]. The mechanism by which 10-HDA and ZnONPs decrease the expression levels of TNF-α, IL-1β, IL-6, IL-8, and NO is likely linked to the inhibition of the NF-κB pathway. The results validated the anti-inflammatory efficacy of 10-HDA and ZnONPs, with the combined treatment demonstrating the most significant ameliorative effects.

Apoptosis represents the third mechanism underlying Pb-induced nephrotoxicity [9]. To enhance our understanding of the mechanisms by which 10-HDA and ZnONPs confer protection to the kidneys against PbAc-induced cell death, we assessed the mRNA expression levels of Caspase-3, Bax, and Bcl-2 in kidney tissue. Caspase-3, Bax, and Bcl-2 are implicated in the mitochondrial-mediated intrinsic apoptotic pathway [59]. The treatment with 10-HDA and ZnONPs exhibited a significant inhibitory effect on PbAc-induced changes in gene expression, particularly the upregulation of caspase-3 and Bax, as well as the downregulation of Bcl-2. These findings are consistent with previously published findings. Abdel-Daim, Alkahtani [62] and Albarakati, Baty [9] have demonstrated that exposure to PbAc leads to a disruption in the apoptotic-regulating proteins (Bax, caspase-3, and Bcl2). Furthermore, it was indicated that the homeostatic balance between anti- and proapoptotic proteins may be disturbed by the Pb substitution of divalent ions, such as calcium, leading to the death of kidney tissues. Lead induces renal apoptosis through oxidative stress and inflammation by disrupting the Bax/Bcl-2 ratio. The imbalance leads to the disruption of mitochondrial integrity and the release of cytochrome c. Caspase enzyme stimulation indicates that Pb activates the apoptotic pathway mediated by mitochondria [62, 89, 90]. Additionally, PbAc-induced elevated NO has been shown to promote apoptosis through caspases-3 and caspases-6, besides the c-Jun N-terminal kinase signalling pathway [59]. The treatment with 10-HDA and ZnONPs significantly modulates pro- and antiapoptotic gene expression in PbAc-induced nephrotoxicity. The administration of 10-HDA and ZnONPs reduced the elevated mRNA expression levels of Bax and caspase-3 while enhancing the mRNA expression levels of Bcl-2 in kidney tissue. This finding is compatible with those of Almeer, AlBasher [59], ATASEVER, and YAĞLI [84], who found that royal jelly resisted cadmium-induced apoptosis and nephrotoxicity through increasing the levels of Bcl-2, an antiapoptotic protein, in rodent models, thus counteracting the toxic effects of cadmium and preventing apoptosis. Moreover, Awadalla, Hussein [88] demonstrated that ZnONPs have a protective effect against renal ischemia/reperfusion injury by downregulating apoptotic genes, specifically caspase-3 and Bax. Additionally, Nrf2/HO-1 signaling activation impeded ROS production and consequently abrogated cell apoptosis [91].

## Conclusion

**Fig. 6:**
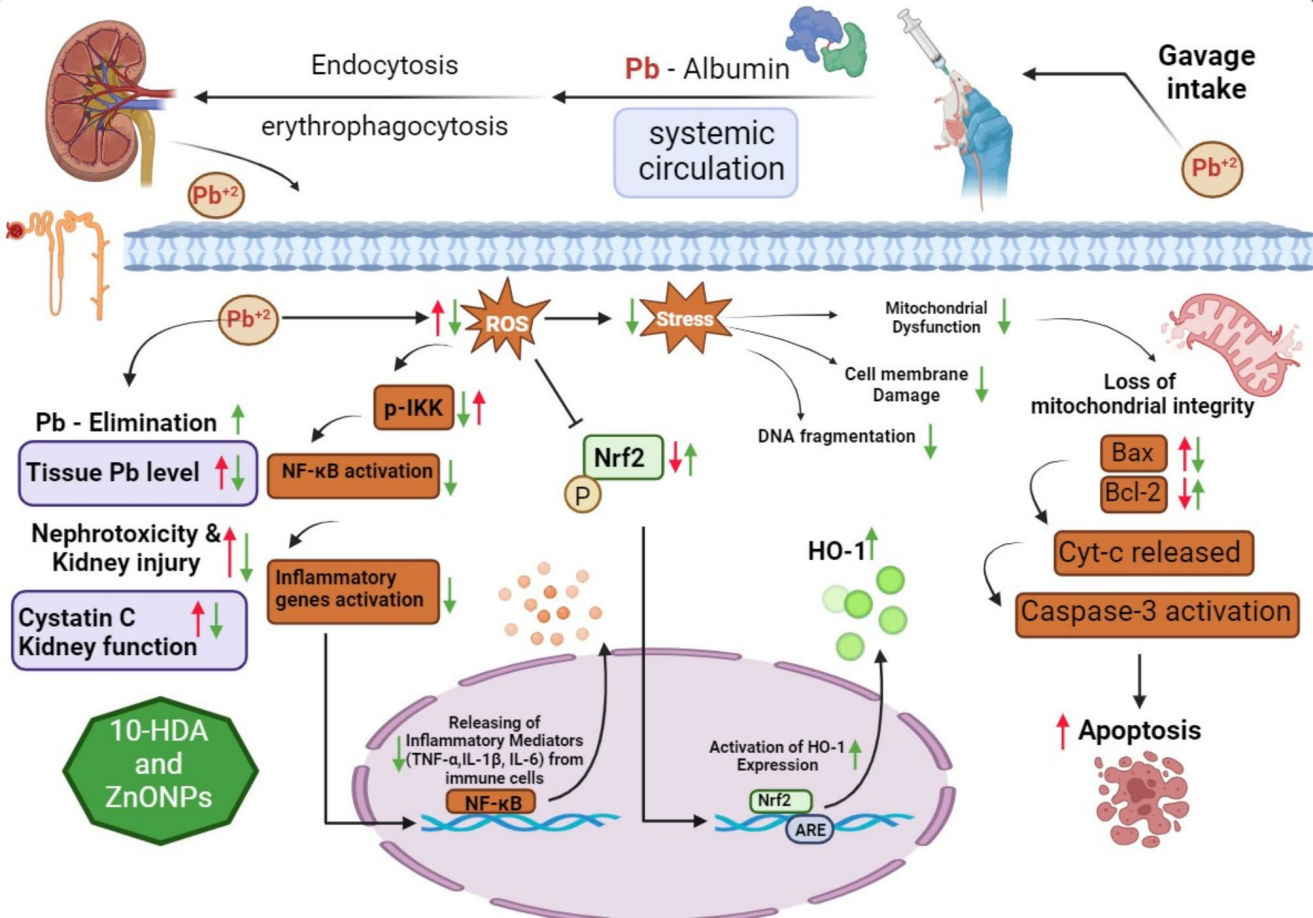
A mechanistic diagram of lead-induced nephrotoxicity and the potential nephroprotective effects of 10-HDA and ZnONPs. Following oral administration, lead enters the bloodstream and binds with albumin. Endocytosis and erythrophagocytosis allow lead to enter the cell. Lead induces oxidative stress through the increased generation of ROS. ROS activate NF-κB signalling through the elevation of phosphorylated IκB kinase (p-IKK) levels, which subsequently facilitates the release of inflammatory mediators, including TNF-α, IL-1β, and IL-6, from immune cells. Oxidative stress induces cellular injury through the compromise of cell membrane and mitochondrial integrity, as well as through DNA damage. The apoptotic pathway is further activated by increasing Bax and Caspase-3 levels while decreasing Bcl-2 levels. Lead concurrently inhibits Nrf2/HO-1 activation, resulting in decreased HO-1 levels responsible for ROS scavenging. This disruption leads to an imbalance in the antioxidant system, a primary contributor to oxidative stress and, ultimately, nephrotoxicity. Treatment with 10-HDA and/or ZnONPs enhances lead elimination from tissue and reduces elevated Cystatin C levels, thereby improving kidney function. Treatment decreases p-IKK, thereby inhibiting inflammatory mediators through the blockade of the NF-κB pathway. Bax and Caspase-3 levels are reduced, whereas Bcl-2 levels increase, inhibiting caspase activation and apoptosis. Additionally, 10-HDA and ZnONPs restore the levels of Nrf2 and HO-1. Lead activity is indicated by red arrows, while ZnONPs and 10-HDA may exhibit a protective effect, as shown by green arrows

PbAc intoxication resulted in the accumulation of lead in the kidneys, alongside elevated kidney function parameters and increased serum levels of cystatin C. The deposition of Pb is associated with increased levels of MDA and GSH, alongside the downregulation of Nrf2 and HO-1 expression levels. Furthermore, PbAc exposure elevated the mRNA levels of pro-inflammatory cytokines and pro-apoptotic markers while decreasing the levels of the anti-apoptotic marker Bcl-2 in the kidney, as shown in Fig. 6. 10-HDA and ZnONPs mitigated lead-induced nephrotoxicity by preventing renal lead accumulation, restoring the oxidant/antioxidant balance, and inhibiting inflammatory and apoptotic signalling. 10-HDA and ZnONPs may serve as effective agents in the treatment of nephrotoxicity induced by heavy metals. Their combination synergized against inflammatory, oxidative stress, and apoptotic responses. This study indicates a benefit for individuals experiencing chronic exposure to elevated lead levels. Further research is required to elucidate the nephroprotective effects of 10-HDA and ZnONPs, particularly regarding the mechanism of lead excretion. Furthermore, this study will serve as a reference and provide a foundation for future investigations in this area.

## Electronic supplementary material

Below is the link to the electronic supplementary material.

Supplementary Material 1

## Data Availability

No datasets were generated or analysed during the current study.

## Abbreviations

10-HDA: 10-hydroxydecanoic acid
10-H2DA: Trans-10-hydroxy-2-decenoic acid
Bcl-2: B-cell lymphoma 2
Bax: Bcl-2-associated X protein
BUN: Blood urea nitrogen
Cas3: Caspase-3
CI: Combination index
EDX: Energy-dispersive X-ray spectroscopy
GFR: Glomerular filtration rate
GSH: Reduced glutathione
GST: Glutathione-S-transferase
HM: Heavy metal
H&E: Haematoxylin and eosin
HO-1: Heme oxygenase-1
IL-1β: Interleukin-1β
IL-6: Interleukin-6
PbAc: Lead acetate
MDA: Malondialdehyde
NPs: Nanoparticles
NO: Nitric oxide
Nrf2: Nuclear factor erythroid 2-related factor-2
Phospho-IKK α/β: Phsopho inhibitor of nuclear factor-κB kinase
ROS: Reactive oxygen species
SEM: Scanning electron microscopy
SOD: Superoxide dismutase
TEM: Transmission electron microscope
TNF-α: Tumour necrosis factor
ZnONPs: Zinc oxide nanoparticles
