# Supplementary figures and images for: Combination of 10-hydroxy-decanoic acid and ZnO nanoparticles abrogates lead acetate-induced nephrotoxicity in rats: targeting oxidative stress and inflammatory signalling
Source: BMC Pharmacol Toxicol. 2025 Mar 25;26:69. doi: 10.1186/s40360-025-00888-1 (PMC11934796; doi:10.1186/s40360-025-00888-1)

Original Raheel

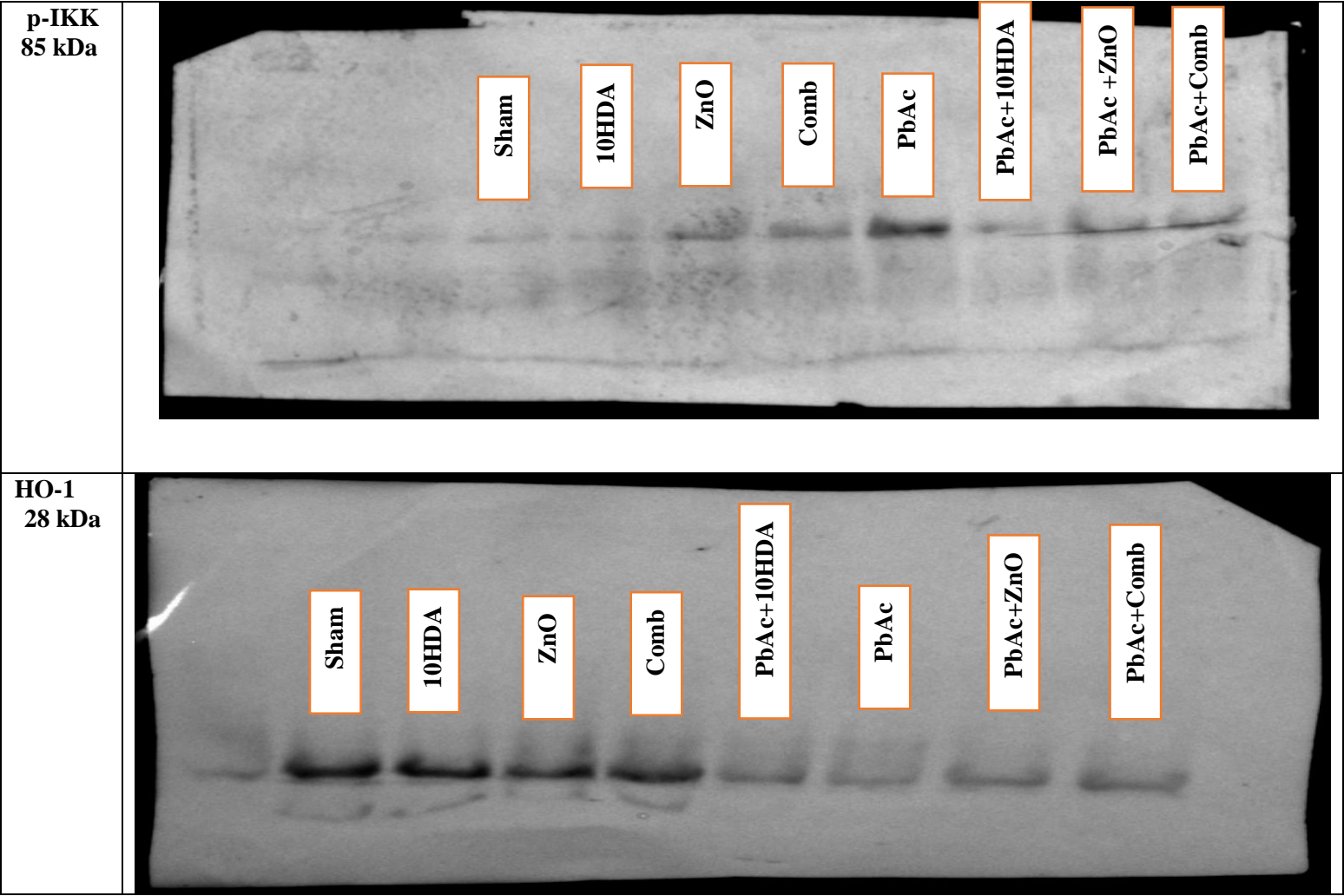

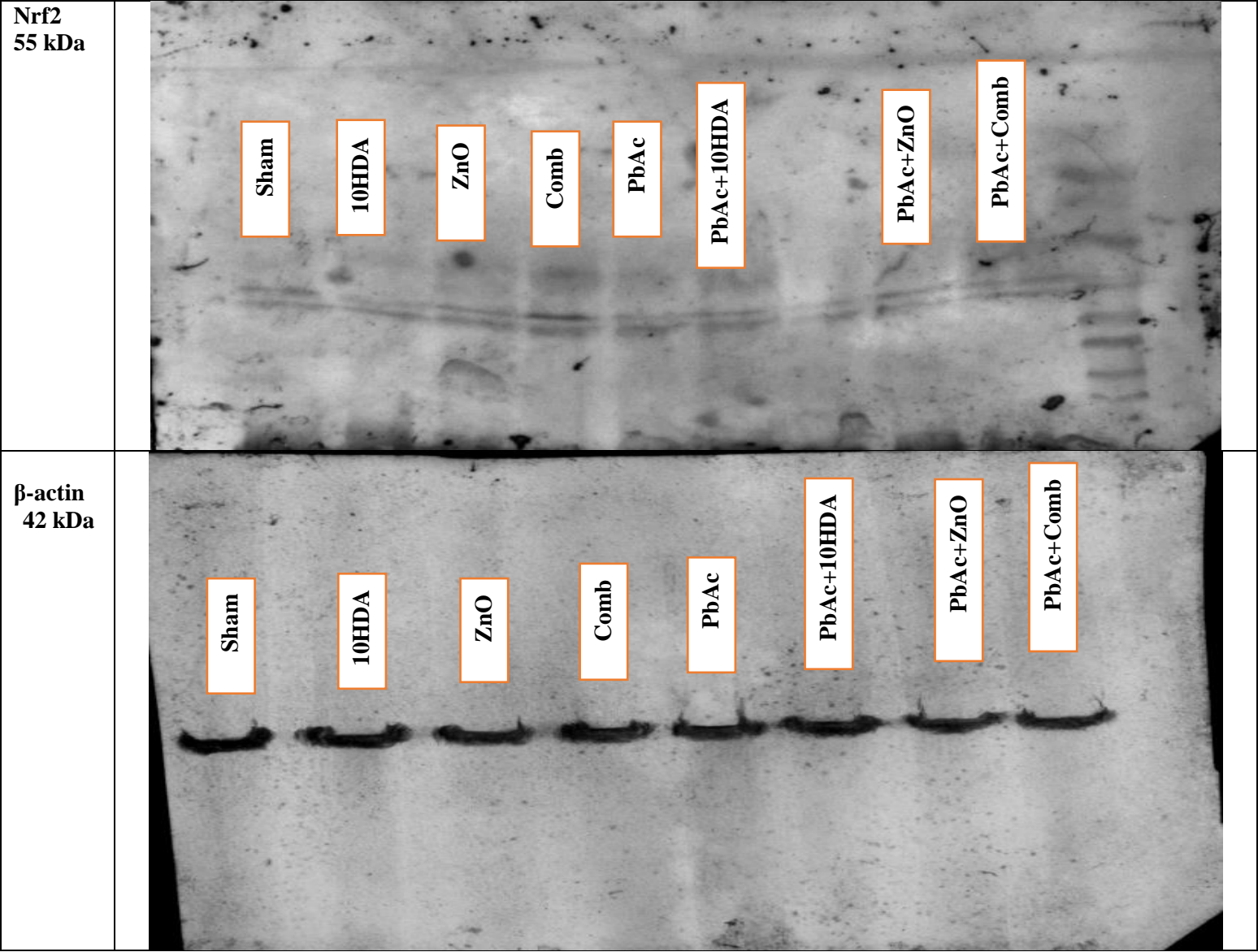

## Original Photo:

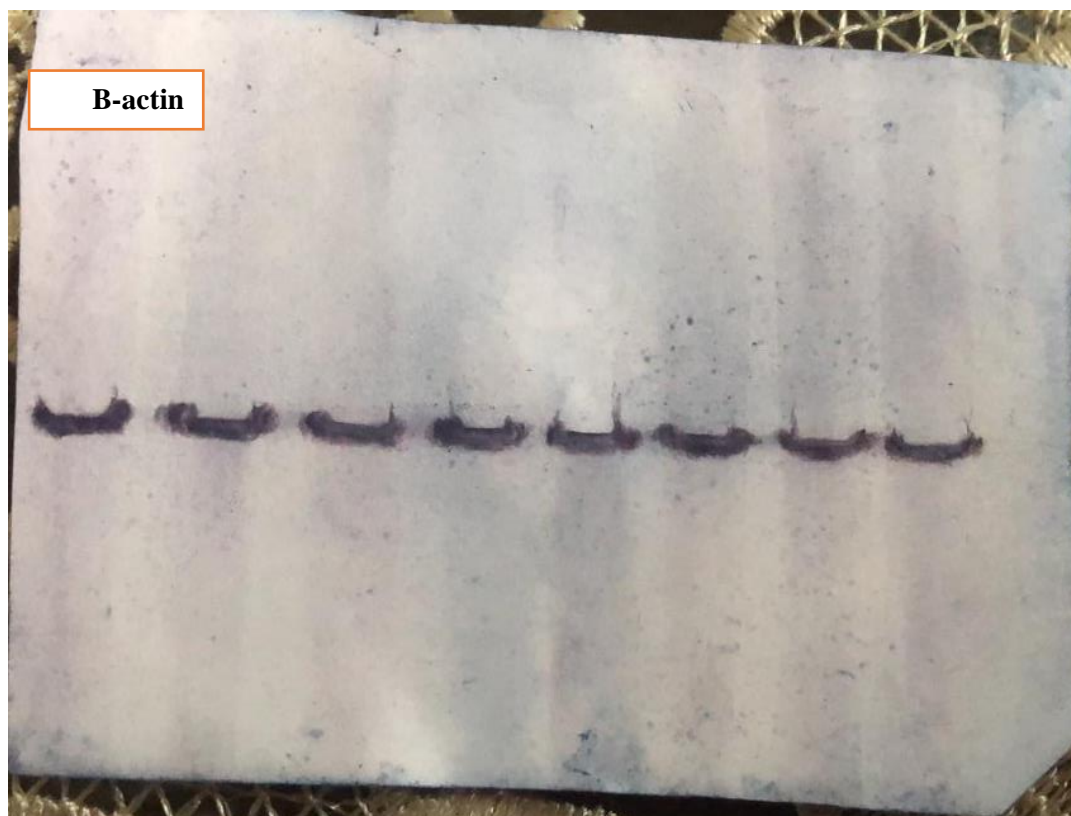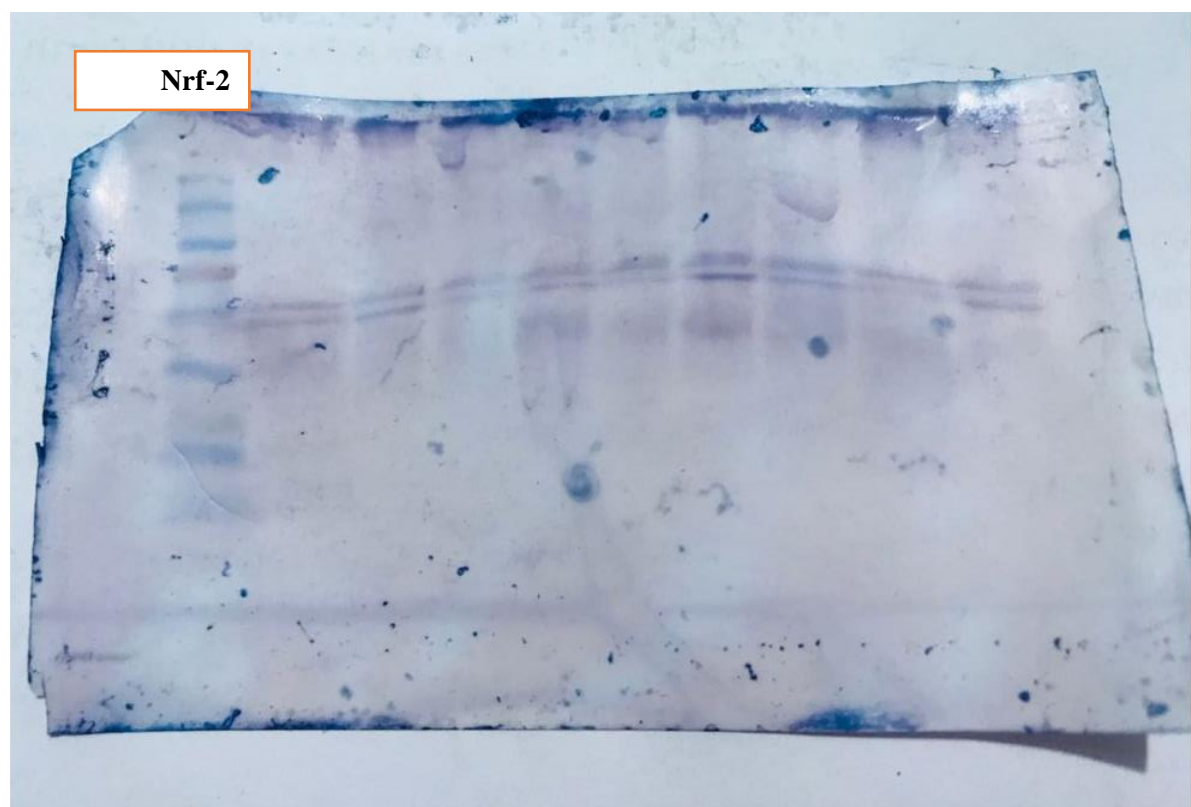

HO-1

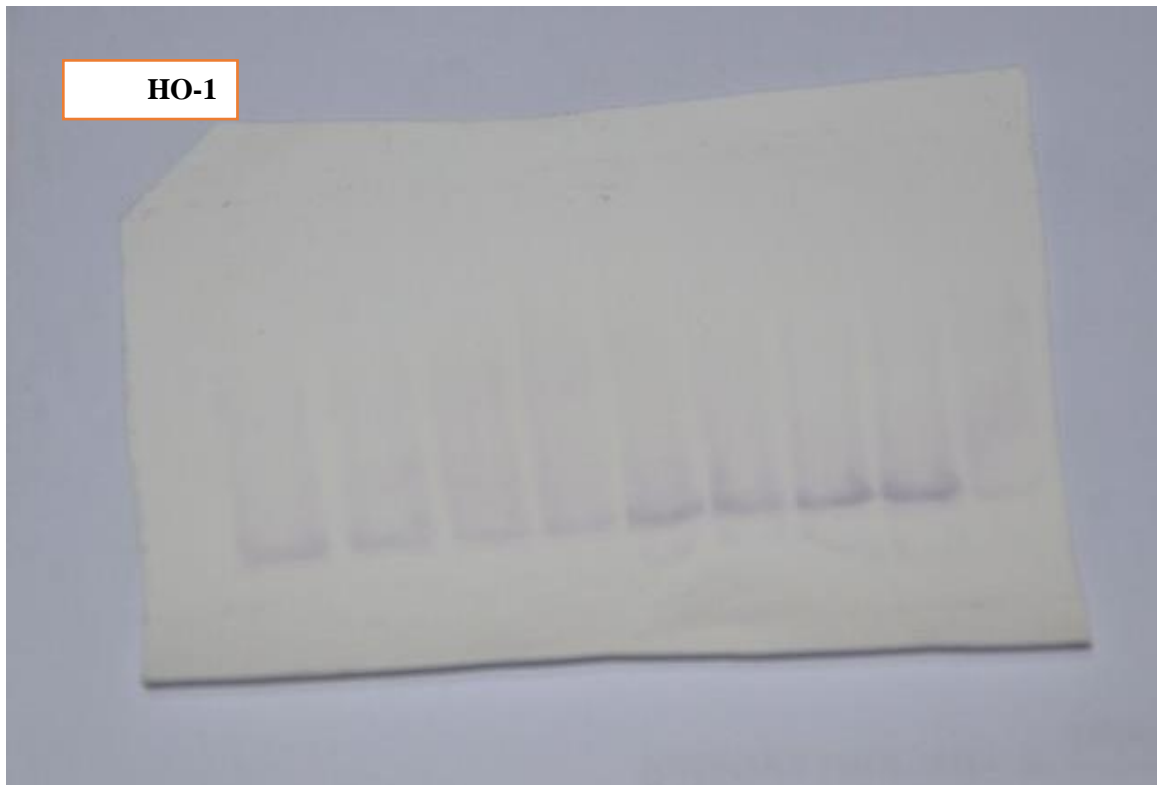

P-IKK

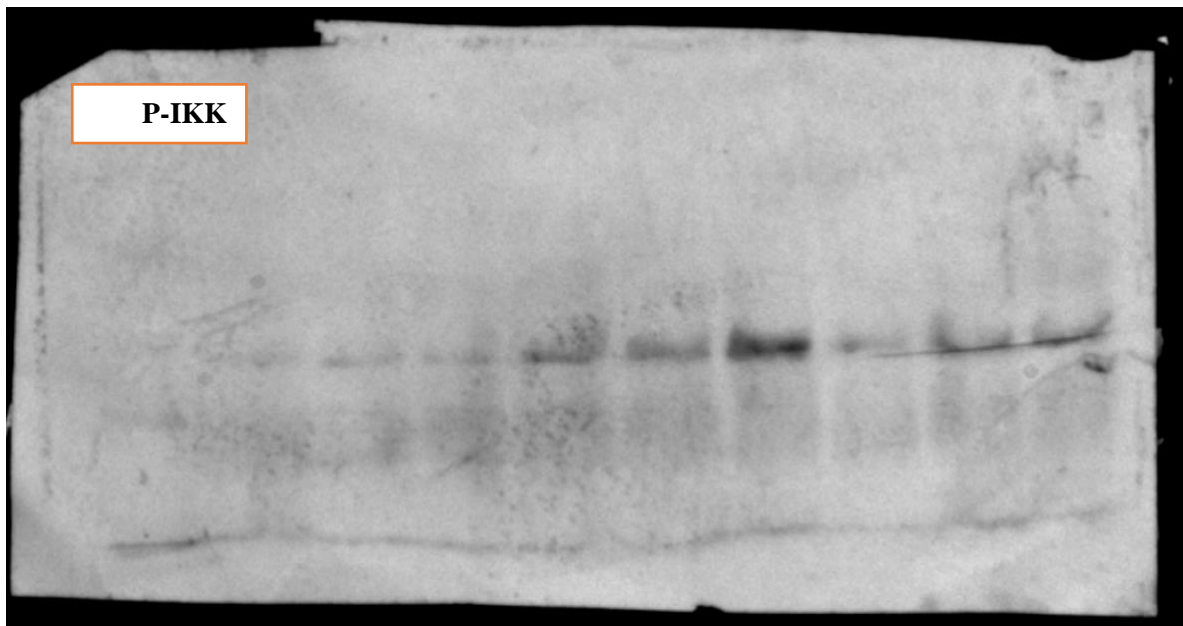

Supplement: Supplementary file 1 — Supplementary Material 1 [file 40360_2025_888_MOESM1_ESM.pdf]
